# Community organization perspectives on COVID-19 vaccine hesitancy and how they increased COVID-19 vaccine confidence: a Canadian Immunization Research Network, social sciences and humanities network study

**DOI:** 10.3389/fpubh.2023.1258742

**Published:** 2023-10-02

**Authors:** Sarah Ashfield, Lorie Donelle, Gina Uppal, Michael A. Bauer, Anita Kothari

**Affiliations:** ^1^Arthur Labatt Family School of Nursing, Faculty of Health Sciences, University of Western Ontario, London, ON, Canada; ^2^Emily Myrtle Smith Endowed Professor of Nursing, Biobehavioral Health and Nursing Science, College of Nursing, University of South Carolina, Columbia, SC, United States; ^3^Department of Computer Science, The University of Western Ontario, London, ON, Canada; ^4^School of Health Studies, Faculty of Health Sciences, University of Western Ontario, London, ON, Canada

**Keywords:** COVID-19, community organizations, vaccines, vaccine hesitancy, trust, vaccine confidence

## Abstract

**Background:**

COVID-19 vaccines play a critical role in reducing the morbidity and mortality associated with SARS-CoV-2 infection and despite vaccine availability, disparities in COVID-19 vaccine uptake among Canadian subgroups exist. Community organizations are uniquely situated to relay important vaccine messaging around all vaccines, understand components of vaccine hesitancy, and facilitate vaccine uptake within the communities they serve. The objective of this research was to solicit community organizations perspectives specific to COVID-19 vaccines and explore strategies of increasing vaccine uptake within their communities.

**Methods:**

A qualitative focus group study was held in the spring of 2021 with 40 community organizations from across the country. Discussions focused on COVID-19 vaccine communication and awareness within their communities, vaccine misinformation, and strategies to increase vaccine acceptance and access. Data were analyzed utilizing thematic and inductive techniques.

**Results:**

Vaccine hesitancy was identified among staff and clients. Vaccine confidence, complacency, convenience, and mistrust in government and authorities were identified as contributors to vaccine hesitancy. Community organizations utilized innovative and novel methods to encourage vaccine uptake and increase vaccine confidence. Leveraging established trusting relationships was key to successful messaging within communities.

**Conclusion:**

Community organizations used innovative methods, built on established trust, to increase vaccine confidence within their communities and among their staff. Community agencies played an important role in COVID-19 vaccine uptake within subgroups of the Canadian population. Community organizations are key public health partners and play a critical role in increasing COVID-19 vaccine confidence.

## Introduction

Vaccination is one of the most effective ways of preventing morbidity and mortality associated with vaccine preventable diseases, yet despite progress vaccination coverage has plateaued (1). The Coronavirus disease 2019 (COVID-19) pandemic has highlighted the important role of public health in disease prevention, detection, and in promoting health. Similar to other infectious disease vaccines, COVID-19 vaccines play a critical role in protecting against severe disease, hospitalizations and death (2). Despite efforts to make COVID-19 vaccination available to all Canadians, disparities in vaccine uptake remain among certain subgroups (3). Mainstream public health messaging is necessary to convey the importance of widespread vaccination to the general public and to dispel disinformation related to vaccines and vaccinations (2, 4). Throughout the COVID-19 pandemic vaccine misinformation and disinformation proliferated (5). Traditionally the term misinformation refers to false or misleading content shared without harmful intent, although the effects can still cause harm, in contrast to disinformation where false information is purposefully spread with the intent to deceive, gain political and/or economical gain (5). COVID-19 misinformation went beyond health aspects and included political responses to the pandemic, origins of the virus, and the severity of the virus (5). These challenges further highlight the need for targeted public health communication strategies to enhance vaccine uptake among ‘under-reached’ and disadvantaged subgroups of Canadians (6, 7). Community organizations play a critical role in facilitating the uptake of vaccine promoting messaging within the diverse populations they serve. There are several terms utilized to describe individuals who have been underserved, marginalized, vulnerable, racialized, colonized, disadvantaged and/or discriminated against, this paper will use the term disadvantaged throughout.

This qualitative research was conducted with the purpose of soliciting community organizations’ perspectives of COVID-19 vaccines and to explore ways in which these organizations work to increase COVID-19 vaccine acceptance among disadvantaged populations. Community organizations targeted in this project included charities, unions, professional associations, community-based organizations, faith groups and social enterprises that provide health care related services to a wide variety of individuals.

## Background

A worldwide COVID-19 pandemic was declared by the World Health Organization on March 11, 2020 (8). This disease spread rapidly throughout the world with cumulative cases exceeding 183 million and more than 4 million deaths worldwide (9). Prior to the COVID-19 pandemic, international public health organizations were concerned about a decrease in uptake of routine childhood vaccinations and increasing vaccine hesitancy resulting in global resurgences of some of the most contagious vaccine preventable diseases (10). The public health response to falling vaccination rates includes the development of social marketing campaigns creating awareness and education around the importance of vaccination (11). The development and implementation of the COVID-19 mass vaccine program is an example of public health response to a public health crisis. Certain groups and populations are at higher risk of SARS-CoV-2 infection the virus that causes COVID-19 including essential workers, those working or living in congregate conditions, group living, Indigenous and remote communities, and marginalized and racialized communities (7).

Communities marginalized through structural factors such as racism, disability, economic disparities, sexual orientation, colonialist health care legacies, and many other structural determinants of health experience inequities in health outcomes including contracting chronic and infectious diseases (7). These inequities in health outcomes extend to an increased risk of SARS-CoV-2 infection, one Canadian example of this is the effect of colonization leading to ongoing racism that continues to impact the healthcare of Indigenous people including higher rates of SARS-CoV-2 infection (7, 12).

In Ontario, one of the Canadian provinces most severely impacted by SARS-CoV-2, infections have taken a disproportionate toll on individuals and families of disadvantaged and racialized urban neighbourhoods (6). Between May 20, 2020, and July 16, 2020, 83% of people in the city of Toronto with reported SARS-CoV-2 infection identified as a racialized group and 51% of reported cases were living in households considered lower income (13). As of August 28, 2021, 67.3% of individuals over the age of 12 years living in Ontario have been fully vaccinated (14). However, there remains a significant proportion of individuals unvaccinated (14).

Vaccine hesitancy—a delay in acceptance, or refusal to get vaccinated despite the availability of vaccine services is framed as a behavior that results from a complex decision-making process; vaccine hesitancy involves three conceptual factors inclusive of confidence, complacency, and convenience (15). Vaccine confidence is defined as trust in the effectiveness and safety of vaccines; the system that delivers them, including the reliability and competence of the health services and healthcare providers; and the motivations of policy makers who decide on the needed vaccines (15). Vaccine complacency occurs when the perceived risks of vaccine preventable disease are low and vaccination is deemed unnecessary (15). Vaccine convenience is when physical availability, geographical accessibility, ability to understand, and appeal of vaccine services affect the decision to be vaccinated (15). Individuals who are vaccine hesitant may accept some vaccines and refuse others, delay some vaccines, or accept vaccines but be hesitant to do so (15). Using the example of COVID 19 vaccines, emerging evidence from the United States, Canada, and the United Kingdom indicates high vaccine hesitancy prevalent among disadvantaged groups (16, 17). Engaging with organizations and community partners that service these disadvantaged communities has been identified as a priority to help address health disparities and better understand vaccine hesitancy (7, 13, 18).

Reported in Canada and around the world were high levels of community transmission and record numbers of intensive care hospitalizations which resulted in widespread and restrictive public health measures to stop the spread of disease, such as stay at home orders, mandatory masking, physical distancing, and limits on social gatherings (19).

For example, Canadians witnessed the largest vaccine program in Canadian history, including the rapid development of mass vaccine clinics and distribution of vaccines to individuals aged 12 years and older. At that time, two mRNA vaccines (Pfizer-BioNTech & Moderna) as well as two viral vector vaccines (AstraZeneca/COVISHIELD & Janssen) were authorized for use in Canada (2). The initial approach to vaccination in Canada was to prioritize individuals at highest risk of hospitalization and death from SARS-CoV-2 infection as well as a first dose approach (2, 20). This strategy prioritized vaccination among adults in order of descending age, those living in community, and long-term care settings as groups of people who had suffered the highest morbidity and mortality from the first wave of COVID-19 and were at high risk for poor outcomes (20). Rapidly evolving evidence around vaccine efficacy against various COVID-19 variants, vaccine side effects, and adverse events following vaccination led to frequent revisions to the guidelines around who should receive what type of vaccines, vaccine mixing, and the associated risks (2, 6).

The ongoing and increasingly higher transmissibility of the SARS-CoV-2 virus created greater urgency for widespread uptake of vaccines to achieve community immunity (21). Community organizations have unique perspectives into the views and challenges their clients face and are increasingly prominent in delivering health and social services to the public (22). Therefore, the purpose of this research study was to better understand community organizations’ perspectives about COVID-19 vaccines and to understand their experience of COVID-19 vaccine acceptance among the people who access their services. The research question guiding this research was: How can third sector community organizations increase COVID-19 vaccine acceptance?

## Methods

Qualitative descriptive research studies are appropriate for understanding a phenomenon, process, or the perspectives of participants and allows for a straightforward description of the experiences and perspectives of participant representatives of community-based organizations regarding COVID vaccines and vaccination processes (23).

### Recruitment & ethical considerations

Recruitment flyers were distributed electronically among an established network of third sector community partners that served a diverse array of individuals. Recruitment messages were also posted on Twitter, LinkedIn, a blog and via email to invite individuals that were accountable for pandemic related communication strategies within these community health organizations to participate in the research study. There was an anticipated sample size of 30–40 participants whose final number was determined through appropriate participant involvement providing rich data and data saturation (24).

Ethics approval was obtained through Western University’s Research Ethics Board (Application #118259). To be included in the study, participants had to be working as a paid employee in a community organization in Canada for at least 1 year, be involved in either communications or management of the organization and be able to communicate in English. Participants were excluded if they were under the age of 18 years.

### Data collection

Semi-structured focus groups were conducted via Zoom communications between April 7, 2021, and May 6, 2021. Zoom was selected as the preferred method of collecting the data as it is an effective method of conducting virtual focus groups (25). Use of virtual focus groups allowed for geographical diversity of participants while maintaining public health restrictions. Data was collected from participants who represent organizations from across Canada. Participants were asked about their experiences communicating with clients about COVID-19 vaccines and vaccine awareness, vaccine communication and misinformation, challenges clients face accessing or understanding COVID-19 vaccine information, and strategies they are using to support and facilitate COVID-19 vaccine access and acceptance. The focus group questions were reviewed for clarity and relevance with one of our community agency partners. Experienced moderators led each session (LD, AK), research assistants took notes during each session (SA, GU), the sessions were audio recorded and professionally transcribed.

### Data analysis

Data collection and thematic analysis occurred concurrently using the “3C’s” framework of vaccine hesitancy guided the data analysis (15). Vaccine confidence, vaccine complacency and vaccine convenience make up the three determinants of vaccine hesitancy in the model adopted by the World Health Organization’s working group on vaccine hesitancy (15). Two researchers iteratively reviewed the transcripts organized using NVivo software. Interview notes and transcripts were read and re-read by two members of the researcher team. Codes were grouped together into sub-themes guided by the 3C’s framework. Once initial coding was completed the codes were reviewed by an interdisciplinary team of researchers. Discrepancies were discussed until a consensus was reached. Data codes were tracked and documented to include exemplar quotes from interview transcripts to demonstrate the meaning of the code. Recruitment of focus group participants continued until no new themes, patterns, or codes were generated from the data and became repetitive in nature.

The thematic analysis process was used to organize the data into themes and subthemes informed by the multidisciplinary research team; that is, themes were deductively generated using the framework and inductively generated from participant responses. Following preliminary data analysis, subthemes and overall findings were discussed among the interdisciplinary research team which consisted of members from the disciplines of public health policy and knowledge translation, computer sciences, and nursing (26). The data analysis process was approached in a systematic and methodical manner to ensure that the results were meaningful and useful (27).

Ensuring trustworthiness throughout the data analysis process was important to ensure that the research findings are acceptable and useful (28). Trustworthiness was determined through credibility established through prolonged engagement (through repeated reading of transcripts and listening to audio transcripts) and dependability (the research process was conducted in logical steps and clearly documented for readers to examine the research process) (28). Through iterative team discussion discrepancies were discussed further until consensus was reached.

## Findings

Forty-one organizational representatives from Ontario, British Columbia, Quebec, and Alberta participated in 11 focus groups. One organization, with a presence in Canada, served a global mandate, 8 organizations were nationally (Canadian) focused, 7 organizations provided services across one province, 1 organization provided services to 2 provinces, and 23 organizations provided local community-focused services within 1 province, and 1 organization did not specify location of services. These organizations serve a wide variety of individuals: the general population, youth and their families, individuals living with cancer or chronic disease, seniors and individuals living with disabilities, immigrants, and low income, marginalized and vulnerable populations. Participating organizations were diverse in terms of their organizational mandate and consequently the pandemic affected their day-to-day operations in a variety of ways. Obligated by the early COVID-19 public health safety strategies, many organizations shifted to virtual or online services. However, some organizations provided essential services that could not be performed virtually and are described below. All organizational representatives reported an increase in their workload to adjust to mandated pandemic restrictions and to ensure their staff and clients remained safe. Yet community organizations employed innovative strategies to enhance uptake of vaccines that are discussed in detail below and summarized in a table in Appendix A.

### Thematic findings

Vaccine hesitancy was the major theme present in the data. Vaccine confidence, vaccine convenience, vaccine complacency and mistrust in the government and large organizations were identified as four sub-themes.

#### Vaccine hesitancy among staff and clients

Vaccine hesitancy was prevalent among staff within the participant organizations as well as within the communities they serve. Several focus group participants reported, “There was hesitancy among our staff” (Participant 11) and “surprisingly our biggest lack of vaccine confidence is with our staff … we have an 80% vaccination rate which is good but it’s still not great … so it tends to be more with the staff” (participant 3).

Vaccine hesitancy was also noted by many participants as prevalent among individuals within the communities that these organizations serve: “there’s a bit of hesitation around the vaccine” (participant 14). Vaccine hesitancy was identified among newcomers to Canada and particularly among the South Asian community, “there’s quite a bit of vaccine hesitancy within these communities [newcomers] because there is quite a bit of hesitancy within the South Asian community right now” (participant 5).

Several organizations identified that accessing vaccine information in a language that individuals could understand was one of the factors relating to vaccine hesitancy among these newcomer groups. “If you do not use a medium or language that people understand, you are not communicating” (Participant 37). Beyond newcomers, individuals who immigrated to Canada years ago may not have the English language skills to understand and make vaccine decisions from mainstream public health messaging. Participant 9: “Maybe they have lived in Canada for 30 years and they still may not speak English.”

Reported reasons for vaccine hesitancy included: fear for personal/family safety, morbidity, and mortality from unknown short and long-term vaccine side effects. Participants reported that individuals accessing their organizations had questions and expressed concerns about vaccine-related ill health, the impact of the vaccine on pregnant and yet-to-be pregnant individuals, concerns related to their children’s health and fear of vaccine-related death. These concerns were identified by participants through the iterative questions they received as reflected below:

“is it going to make me sick” (Participant 38); “there was hesitancy and once you find out one stat about one person dying because of one vaccine, there’s so much hesitancy because of that one thing”; (Participant 9); “is it safe for me, I may become pregnant, you know is it going to be safe for me if I’m going to have a baby” (Participant 38).

The rapid development of vaccines “how come they were approved so quickly, what steps did they skip, can we trust it, is it safe” (Participant 38) and concern that messenger RNA vaccines would alter an individual’s DNA “is it going to change my DNA” (Participant 38) also contributed to vaccine hesitancy among individuals served by community organizations.

#### Vaccine confidence

*Vaccine confidence* is defined as trust in (i) the effectiveness and safety of vaccines; (ii) the system that delivers them, including the reliability and competence of the health services and health professionals and (iii) the motivations of policy makers who decide on the needed vaccines (15). Vaccine confidence was identified as a factor in vaccine hesitancy among staff within some organizations. Participant 4 reported “more unexpected was that there continued to be a lack of confidence among staff. We had offered to provide training and education, webinars, to staff and early on executive directors declined, they said there’s really not much interest, we are not concerned about that, but then when they started to offer the vaccine to staff, they did encounter challenges.”

One organization (Participant 3) that served older adults in a long-term care setting identified a lack of vaccine confidence among staff that they did not see among the long-term care residents. The participant reported excellent uptake of vaccines among clients living in long-term care settings in comparison to organizational staff stating that, “our biggest lack of confidence is with our LTC staff … so it tends to be more with the staff than it is with the clients and residents, and that’s speaking for community and LTC.”

Rapidly changing and conflicting information on vaccine side effects was also identified as a factor in vaccine confidence. Changing guidelines about the risks and side effects of the COVID-19 AstraZeneca (AZ) vaccine caused significant fear and anxiety among individuals, some already with compromised health; “So the panic I had within the group … oh no I do not want AZ” (participant 39), and “the whole issue about AZ vaccine in causing blood clots because some of the medication in cancer does cause blood clots” (participant 12). Participants found it challenging to support the information needs of clients related to their concerns about vaccine side effects. Of note was the concern about vaccine related risk of blot clot and one participant (7) stated that “… we always find the science is moving very quickly, but the science is not moving fast enough for them to answer some of the questions that they [clients] have” (participant 7).

Evolving information around the prevalence and risk of Vaccine Induced Thrombotic Thrombocytopenia after vaccination with AZ vaccine among different age groups caused many individuals to fear this specific vaccine brand. Participants reported a lack of transparency around the risk with this particular vaccine causing some clients to emphatically refuse vaccination with AZ vaccine. “They do not want Astra Zeneca because of the media flip flopping” (participant 28) … they specifically want Pfizer and not Astra Zeneca” (participant 33).

##### Trust/mistrust

Participants noted a lot of government and large corporation mistrust within communities. “It’s very difficult to share government resources when there’s already so much government mistrust … We have a lot of clients from very vulnerable communities who do not trust the system.” (Participant 7) Community organizations heard from their clients that they did not trust vaccine information coming from large government and health related corporations. Participant 21 reported; “quite a bit of trust has been sort of lost, or there’s been back and forth on trust when it comes to large organizations” and “I noticed there was a lot of distrust around the World Health Organization.” Participants reported that their clients expressed concern about being lied to as reflected by the following: “… one day they say one thing, and the next day the opposite, so people are wondering are scientists lying, who can we actually trust, who can we believe” (Participant 21).

Vaccine prioritization of specific groups of individuals (Indigenous, older adults Canadians) by provincial and federal bodies that was intended to safeguard individuals who were most vulnerable to SARS-CoV-2 infection appeared to have the opposite effect and served to further erode trust in the government. Participant 7 reported that vaccine-priority groups felt as though they were identified for experimentation purposes. Senior groups and Indigenous communities wondered, “why are we going first” “are we lab rats” “do they want to see what happens to us first.” One participant identified the historical legacy of unethical experiments conducted by governments with racialized groups of people may have fueled some of this mistrust among individuals. “Some of them were talking about Tuskegee, and [mistrust] is so deep and so dense” (Participant 7).

Community organizations found that maintaining trusting relationships was key to providing science-informed guidance and keeping their clients/communities safe during the pandemic. “The strategies employed by our agencies were very much about building and maintaining trust and using the established trust that they have” (Participant 4). Science-informed guidance was described by one participant as having: “…accurate scientific information in conjunction with people in the community, at the point where the information is going to be accurately represented to them in a way that is accessible and understandable” (Participant 21).

Beyond maintaining trust, several participants identified that building from a foundation of trust within their communities was key to increasing vaccine confidence, “the trust piece is huge.” Another participant identified the integration of COVID 19 information into already established programming was helpful and doing things such as:

“… developing presentations that people could attend into our existing community program groups that we already had, we intentionally had multiple workshops around vaccines and we would have our nurses who are part of the newcomer clinic present. So, there was already some trust there, when it came to it they knew the nurse” (Participant 11).

Previously established relationships with community workers, health promotors, peer leaders, and nurses were specifically identified as helpful in supporting vaccine decision-making. This established trust was identified as key in vaccine uptake in some specific populations, “we have actually gotten really good uptake from people who are experiencing homelessness which is great, and I think that part of it is just the trust we have been able to develop” (Participant 11).

The use of empathetic and compassionate listening was also identified as important to developing and maintaining trusting relationships among community members. “I try to lead with compassion and empathy and relate to what it is they they are going through” (Participant 21). Participants reported that listening to individuals’ expressed frustrations, challenges, fears, anxieties, and disappointments was an important part of vaccination communication.

“I don’t want to shut them down because to me that’s quite dismissive, and the way in which we will share with them, we want to say we’re having a dialogue, so if you’re going to shut down their concerns why are they going to listen to what you have to say” (Participant 21).

#### Vaccine convenience

Vaccine *convenience* is when physical availability, ability to understand (language and health literacy) and appeal of immunization services influence vaccine uptake (15). Participants reported that their organizations used innovative and novel approaches within their communities to enhance vaccine convenience. One example of one organization’s efforts to increase vaccine appeal was demonstrated through hosting a virtual games night, Participant 10 stated:

“it’s for the community and we’ve done so far 3 or 4 of them with great success, the community loves them we honestly in December when she had this idea was supposed to be a one off and so it was supposed to be let’s do this Zoom trivia night where we invite people to see how much they know about this vaccine that’s coming, but we never thought it would be this recurring event.”

Clients of this community organization provided feedback that participating in this event provided them with accurate vaccine information and moved them from being vaccine hesitant to vaccine acceptant. Some individuals credited this event to changing their decision to be vaccinated against COVID-19, “she told us yesterday that it was only because of our last vaccine trivia night that she was convinced that she should go do the vaccine.” (Participant 10).

Another innovative approach was the use of community ambassadors to increase vaccine convenience. “We have this vaccine engagement community ambassador project where we recruit peers who are resident leaders, who are newcomers themselves, who speak that language and live in those priority neighbourhoods” (Participant 2). Community ambassadors used a variety of approaches to encourage vaccination; participants reported they would, “knock on doors and engage neighbours and try to help them book vaccine appointments; so it really is like doing grass roots health promotion through these peer supporters” (Participant 2). The community ambassadors engaged in one-on-one verbal communication, posted on prominent social media platforms, went door to door to educate about vaccine opportunities, and/or interviewed and presented on various media platforms. Participants also identified that alternative social media platforms were successfully used to convey information in various community groups. WhatsApp (a social media communication app) was a prominent method of communication among some refugee groups and migrant farm workers; “WhatsApp is the primary way to reach migrant farm workers who do not maybe even have cell access but they have got WIFI on the farm” (Participant 4) and “A lot of Syrian refugees that we work with, the only information source is WhatsApp” (Participant 19). These platforms were key to disseminating accurate information about COVID-19 vaccines and how to access them.

Vaccine convenience within the 3C’s model also identifies language as a key component of vaccine convenience (15). Some organizations recognized the relevance of health literacy as an important determinant of health. “We had to take into consideration that a lot of our clients may not be receiving traditional forms of communication about COVID, whether it be language barriers, or a lack of access to the internet or traditional news media” (participant 11) and “if you do not use a medium or a language that people understand you are not communicating” (Participant 37). Similarly, Participant 11 reported that:

“We did a much more concerted effort with our ethno-racial communities … we made a concerted effort in translating these [vaccine] presentations to some of the languages in the communities we serve that are not necessarily the basic languages that you would see in public health …. We do the [vaccine] presentation but also have a voice recording over it so that they could follow along if they didn’t have the literacy level to understand the written form of it” (Participant 11).

Another participant identified focused messaging for their clients

“… we do create messaging specifically for young people and we really try to pull out the pertinent information and do a lot of plain language review … we’ve really leaned on that health literacy team’s expertise to support us in creating communication” (Participant 6).

#### Vaccine complacency

Some participants identified vaccine complacency among their organizational staff and within the public they serve. Vaccine *complacency* occurs where perceived risks of vaccine-preventable diseases are low and vaccination is not deemed necessary (15). Canada’s vaccine rollout plan identified individuals working with vulnerable populations as candidates for priority COVID-19 vaccine access to protect those most vulnerable to the disease (2). However, despite vaccine availability, some individuals did not act upon the opportunity to get vaccinated and reported wanting to wait until more of the population was vaccinated prior to getting their own vaccine, “I’m just going to wait till everyone else does it and see what happens to them (participant 27).”

Other ways vaccine complacency was seen was through doubt and non-belief that SARS-CoV-2 infection was of concern. Participant 7 reported, “I’ve dealt with a couple skeptics who do not believe that it is anything more than a flu.”

## Discussion

Community organizations providing services to a wide array of groups across Canada participated in focus groups regarding perspectives on COVID-19 vaccines within those communities. A wide variety of individuals were represented by the participating organizations including: the general population, youth and their families, individuals living with cancer or chronic disease, seniors and individuals living with disabilities, immigrants, and low income, and marginalized populations. Vaccine hesitancy was reported among staff, volunteers, and clients within many of the participant organizations. Three determinants of vaccine hesitancy, *confidence, convenience, and complacency,* previously identified within the population in relation to other well-established vaccines were applicable to COVID-19 vaccines (15).

The organizations in this study identified areas of need within their various communities and independently took on COVID-19 vaccine promotion and education. These findings are consistent with research conducted in Amsterdam and New York City where community organizations identified and developed solutions to unique challenges in the form of pandemic response among communities marginalized by race, immigration status, religion, social class, and gender (29). As valuable public health partners, the community organizations in our study demonstrated innovative methods of vaccine education and supported vaccine confidence within their communities. The importance of a trusting relationship between those providing vaccine education and individuals making vaccine decisions has been well established in the literature (30). Building on a foundation of established trust the organizations developed novel approaches to engaging in health promotion and addressing vaccine hesitancy within their communities. Some examples of the novel approaches to maintain and enhance trust included the use of community-based vaccine ambassadors, tailored vaccine promotion on social media platforms specific to subgroups of the population and facilitated communication and education through various methods of translation.

The use of community ambassadors was an innovative and personalized approach to public health vaccine messaging different than widespread public health vaccine messaging (31). This personalized approach is consistent with the findings of another study that used parents as vaccine advocates as a part of a community-based approach to reduce vaccine hesitancy (32). Further research that evaluates the role of a community ambassador and the impact on vaccine confidence and vaccine uptake within communities is warranted.

Mistrust in government and large organizations was identified as a factor in vaccine confidence among some participants and their clients within this study. These findings are consistent with preliminary non population-based research studies from other countries (Norway and the United Kingdom) that have also identified trust a variable in COVID-19 vaccine hesitancy (33, 34). Trust is a well-established component of vaccine confidence as one of three determinants of vaccine hesitancy (15).

While trust is a well-established component of vaccine confidence, the COVID-19 pandemic has demonstrated the ongoing health disparities that affect ethnically diverse populations across Canada (7, 35). Populations that have historically experienced health and social inequities were at greater risk of contracting COVID-19 and having more severe disease (7). Historical injustices to Indigenous, racialized, and vulnerable individuals have created justified mistrust within the government (36, 37). The mistrust extends to healthcare providers as Indigenous people and other disenfranchised groups with Canada experience persistent and systemic racism and its impact on healthcare (12). Contemporary examples of how Indigenous, black, racialized, and low-income individuals experience the healthcare system leads to a lack of confidence and trust in the system (38). Our findings are consistent with other research that evaluated attitudes and perceptions around human papillomavirus and influenza vaccines and found mistrust prevalent among racial and ethnically diverse populations (39). Understanding vaccine confidence among these populations is critical as they are among the most vulnerable to COVID-19 and vaccination is key in protecting these individuals from associated morbidity and mortality.

More work needs to be done to further understand how healthcare providers and government systems can work to build trust within these diverse groups of clients served by these community organizations. The important work that these community agencies are doing within their communities demonstrates that a foundation of trust is critical to forming long lasting relationships. Listening and giving voice to these important agencies may provide valuable knowledge that could help inform the healthcare system on effective techniques for establishing and building trust.

### Limitations

Research involving individuals’ response to an emerging health crisis such as the COVID 19 pandemic is a fluid and evolving process. This research was conducted within a specific time frame within the pandemic therefore, findings should be interpreted with these specifics. A sample of community organizations from across the country were included in this study, therefore findings may not represent all community organizations within Canada. This study was conducted in the English language and may not include the perspectives of community organizations that communicate in other languages. The 3C’s framework of vaccine hesitancy was utilized to guide data analysis however, other frameworks may highlight the findings from different perspectives.

### Conclusion and considerations for further research

Findings from this study suggest that vaccine hesitancy was evident among community-based organizations across Canada, their staff, volunteers, and the people they serve. The 3C’s framework of Vaccine Hesitancy is applicable to these new vaccines. Research that builds on these findings would contribute to our understanding of how healthcare providers and government systems can work to build trust within these specific subgroups of the Canadian population. The important contribution of these community agencies in support of vaccine uptake reinforces the need for a foundation of trust perceived as critical to successfully reaching under-served individuals. Public health organizations may benefit from establishing strong partnerships with community-based organizations to leverage the foundation of trust already established as a way to increase vaccine confidence regarding all forms of vaccine preventable diseases. Identifying, funding, and partnering with these organizations could be instrumental in combatting vaccine hesitancy, and provide safe, ethical, and culturally appropriate healthcare to equity deserving individuals.

### Contributions to knowledge

What does this study add to existing knowledge?

- Vaccine hesitancy exists towards the novel COVID-19 vaccines.
- The 3C’s model of vaccine hesitancy is applicable to the novel COVID-19 vaccines.
- Community agencies were important public health ambassadors and used novel methods to increase vaccine confidence.

What are the key implications for public health interventions, practice, or policy?

- Identifying and funding community agencies within Canada is instrumental in providing safe, ethical, and culturally appropriate healthcare to disadvantaged individuals.
- Learning from community organizations may provide valuable knowledge on effective techniques to foster trust as a method to increased vaccine confidence and decrease vaccine hesitancy.
- While focused on COVID-19 immunizations, these findings may translate into supporting uptake of other mandatory vaccinations.

## Data availability statement

The raw data supporting the conclusions of this article will be made available by the authors, without undue reservation.

## Ethics statement

The studies involving humans were approved by Western University Research Ethics Board Application 118259. The studies were conducted in accordance with the local legislation and institutional requirements. The participants provided their written informed consent to participate in this study.

## Author contributions

SA: Data curation, Formal analysis, Investigation, Writing – original draft, Writing – review & editing. LD: Conceptualization, Data curation, Formal analysis, Investigation, Methodology, Supervision, Validation, Writing – original draft, Writing – review & editing. GU: Data curation, Investigation, Project administration, Writing – review & editing. MB: Conceptualization, Methodology, Validation, Writing – review & editing. AK: Conceptualization, Data curation, Funding acquisition, Investigation, Methodology, Project administration, Resources, Supervision, Writing – review & editing.

## Appendix A

Methods to enhance vaccine acceptance among community organizations.

**Table 1:**

Building and maintaining trusting relationships	Communicating in various languages and dialects
Use of traditional communications methods	Providing information in creative and innovative ways
Use of community ambassadors	Translation of vaccine information
Identifying local community needs	Identifying and correcting vaccine misinformation*

*Details of these methods are described in the findings section of the manuscript above.
